# Analysis of heterologous expression of *phaCBA* promotes the acetoin stress response mechanism in *Bacillus subtilis* using transcriptomics and metabolomics approaches

**DOI:** 10.1186/s12934-024-02334-z

**Published:** 2024-02-21

**Authors:** Tao Li, Haixiang Li, Lei Zhong, Yufei Qin, Gege Guo, Zhaoxing Liu, Ning Hao, Pingkai Ouyang

**Affiliations:** College of Biotechnology and Pharmaceutical Engineering, State Key Laboratory of Materials-Oriented Chemical Engineering, Jiangsu National Synergetic Innovation Center for Advanced Materials (SICAM), Nanjing Tech University, Nanjing, 211816 China

**Keywords:** Acetoin, Polyhydroxybutyrate (PHB), Stress tolerance, Transcriptomic, Metabolomic

## Abstract

Acetoin, a versatile platform chemical and popular food additive, poses a challenge to the biosafety strain *Bacillus subtilis* when produced in high concentrations due to its intrinsic toxicity. Incorporating the PHB synthesis pathway into *Bacillus subtilis* 168 has been shown to significantly enhance the strain’s acetoin tolerance. This study aims to elucidate the molecular mechanisms underlying the response of *B. subtilis* 168-*phaCBA* to acetoin stress, employing transcriptomic and metabolomic analyses. Acetoin stress induces fatty acid degradation and disrupts amino acid synthesis. In response, *B. subtilis* 168-*phaCBA* down-regulates genes associated with flagellum assembly and bacterial chemotaxis, while up-regulating genes related to the ABC transport system encoding amino acid transport proteins. Notably, genes coding for cysteine and d-methionine transport proteins (*tcyB*, *tcyC* and *metQ*) and the biotin transporter protein *bioY*, are up-regulated, enhancing cellular tolerance. Our findings highlight that the expression of *phaCBA* significantly increases the ratio of long-chain unsaturated fatty acids and modulates intracellular concentrations of amino acids, including l-tryptophan, l-tyrosine, l-leucine, l-threonine, l-methionine, l-glutamic acid, l-proline, d-phenylalanine, l-arginine, and membrane fatty acids, thereby imparting acetoin tolerance. Furthermore, the supplementation with specific exogenous amino acids (l-alanine, l-proline, l-cysteine, l-arginine, l-glutamic acid, and l-isoleucine) alleviates acetoin’s detrimental effects on the bacterium. Simultaneously, the introduction of *phaCBA* into the acetoin-producing strain BS03 addressed the issue of insufficient intracellular cofactors in the fermentation strain, resulting in the successful production of 70.14 g/L of acetoin through fed-batch fermentation. This study enhances our understanding of *Bacillus*’s cellular response to acetoin-induced stress and provides valuable insights for the development of acetoin-resistant *Bacillus* strains.

**Supplementary Information:**

The online version contains supplementary material available at 10.1186/s12934-024-02334-z.

## Introduction

Acetoin (3-hydroxy-2-butanone) stands as a versatile platform chemical extensively employed in diverse industries such as food, cosmetics, and plant growth promotion [1, 2]. Its industrial production traditionally relies on environmentally unfriendly chemical synthesis, raising safety concerns when used as a food additive [3, 4]. Consequently, there is a growing interest in adopting more sustainable and safer microbial fermentation methods [5].

Various strains, obtained through natural screening and metabolic engineering modifications, have been explored for fermentative acetoin production, encompassing *Bacillus subtilis*, *Bacillus licheniformis*, *Corynebacterium glutamicum*, *Saccharomyces cerevisiae*, *Escherichia coli*, *Candida glabrata* and *Clostridium acetobutylicum* [6–8]. Among these, *B. subtilis*, a natural acetoin producer, distinguishes itself due to its biosafety profile and well-established genetic manipulation systems, making it the preferred strain for acetoin fermentation [9]. In comparison to expression systems such as *Escherichia coli*, *Saccharomyces cerevisiae*, and *Corynebacterium glutamicum*, the *B. subtilis* expression system is considered safer (GRAS, generally recognized as safe) for producing food additives like acetoin and tetramethylpyrazine [10]. Moreover, the rapid growth of *B. subtilis*, its robust industrial fermentation performance, and its ability to metabolize various sugars make it a promising option for the cost-effective acetoin production [11, 12]. In recent years, the application of synthetic biology strategies and effective tools has further enhanced *B. subtilis*’s capability to produce various chemicals and enzymes [13]. These competitive advantages position *B. subtilis* as an ideal host for acetoin production.

Previously, studies have underscored the toxicity of acetoin to microbial cells, including its inhibitory effects on *Staphylococcus aureus* cultures and *Bacillus subtilis* 168 growth [14, 15]. Overcoming this bottleneck in acetoin production necessitates a thorough understanding of the resistance factors and tolerance mechanisms of *Bacillus* to acetoin.

One promising strategy for enhancing strain resistance involves introducing the polyhydroxybutyrate (PHB) synthesis pathway in host bacteria. Studies by Hye-Rim Jung, Mathiyazhagan Narayanan, and Hun-Suk Song have demonstrated introducing PHB synthesis genes in various bacteria can increase tolerance to different stressors and enhance production [16–18]. PHB serves as a carbon and energy storage polymer within microbial cells, offering protection against UV stress, starvation, heat stress, and osmotic shock [19, 20]. Given that introduction of the PHB synthesis pathway in *Escherichia coli* has improved tolerance to organic solvents, this strategy holds promise for addressing acetoin tolerance [18].

In this study, we introduced the polyhydroxybutyrate (PHB) synthesis pathway into *B. subtilis* 168, resulting in a significant enhancement of acetoin tolerance, with the acetoin tolerance concentration reaching more than 80 g/L. To illuminate the underlying tolerance mechanisms, we employed transcriptomics and metabolomics to analyze relevant metabolic pathways and differentially expressed genes. Our analysis revealed that the introduction of the PHB synthesis gene increased the proportion of long-chain unsaturated fatty acids in the cell membrane of *B. subtilis* 168. Simultaneously, it elevated the intracellular concentration of specific amino acids associated with tolerance, as well as biotin concentration. To assess the impact of the improved tolerance of *B. subtilis* to acetoin on fermentation, we used *B. subtilis* 168 as the starting strain, knocked out genes related to the competitive pathway (*ldhA*) and the decomposition pathway of the acetoin synthesis pathway (*acoA*, *bdhA*), and integrated key enzymes of the acetoin synthesis pathway (*alsSD*, *yodC*), successfully constructing the acetoin production strain BS03. Furthermore, incorporating PHB synthase into the acetoin-producing strain BS03-*phaCBA* successfully addressed the issue of insufficient intracellular cofactors in the fermentation strain, ultimately resulting in the production of 70.14 g/L of acetoin through fed-batch fermentation.

## Result

### Impact of acetoin stress on the growth of *B. subtilis *168-pMA5 and *B. subtilis *168-*phaCBA*

**Fig. 1:**
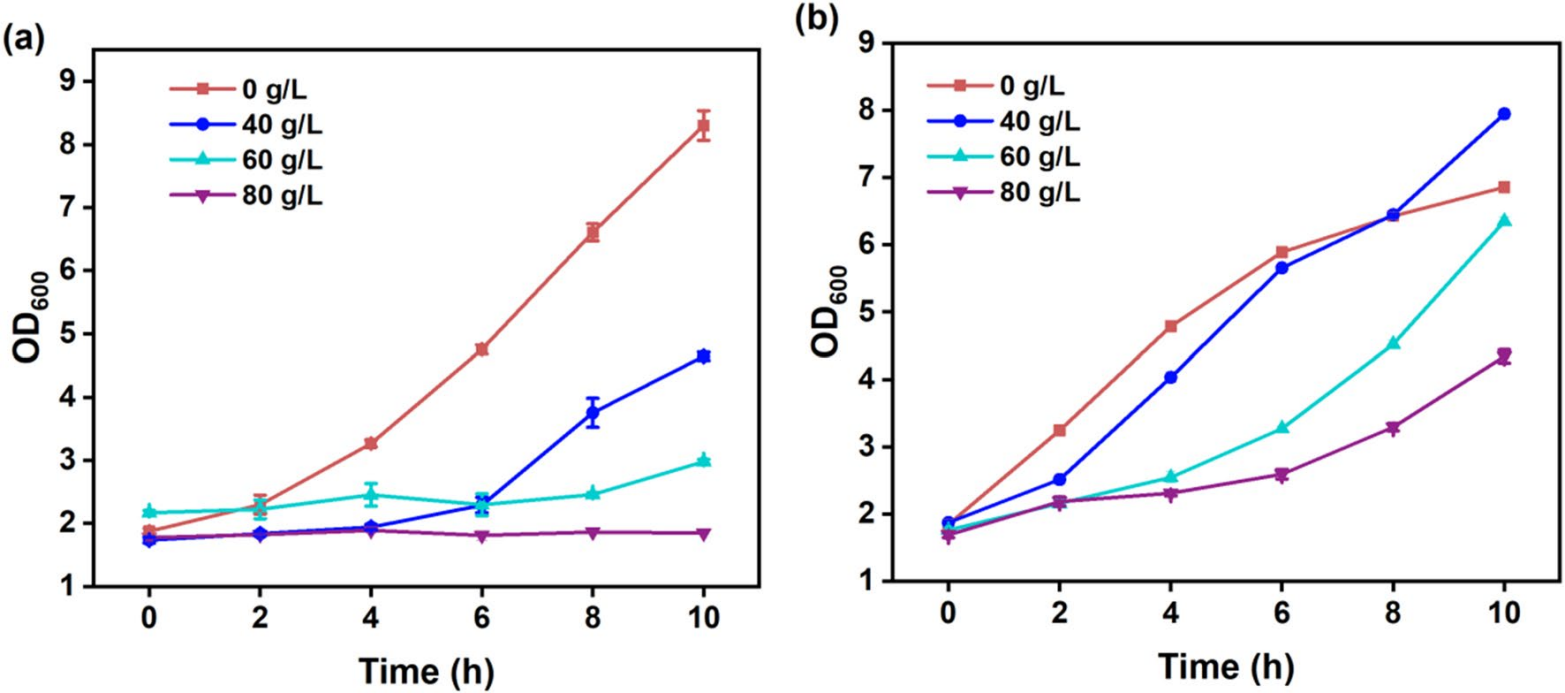
Effects of different acetoin treatments (0 g/L, 40 g/L, 60 g/L, 80 g/L) on the growth of *B. subtilis* 168-pMA5 (**a**) and *B. subtilis* 168-*phaCBA* (**b**)

**Fig. 2:**
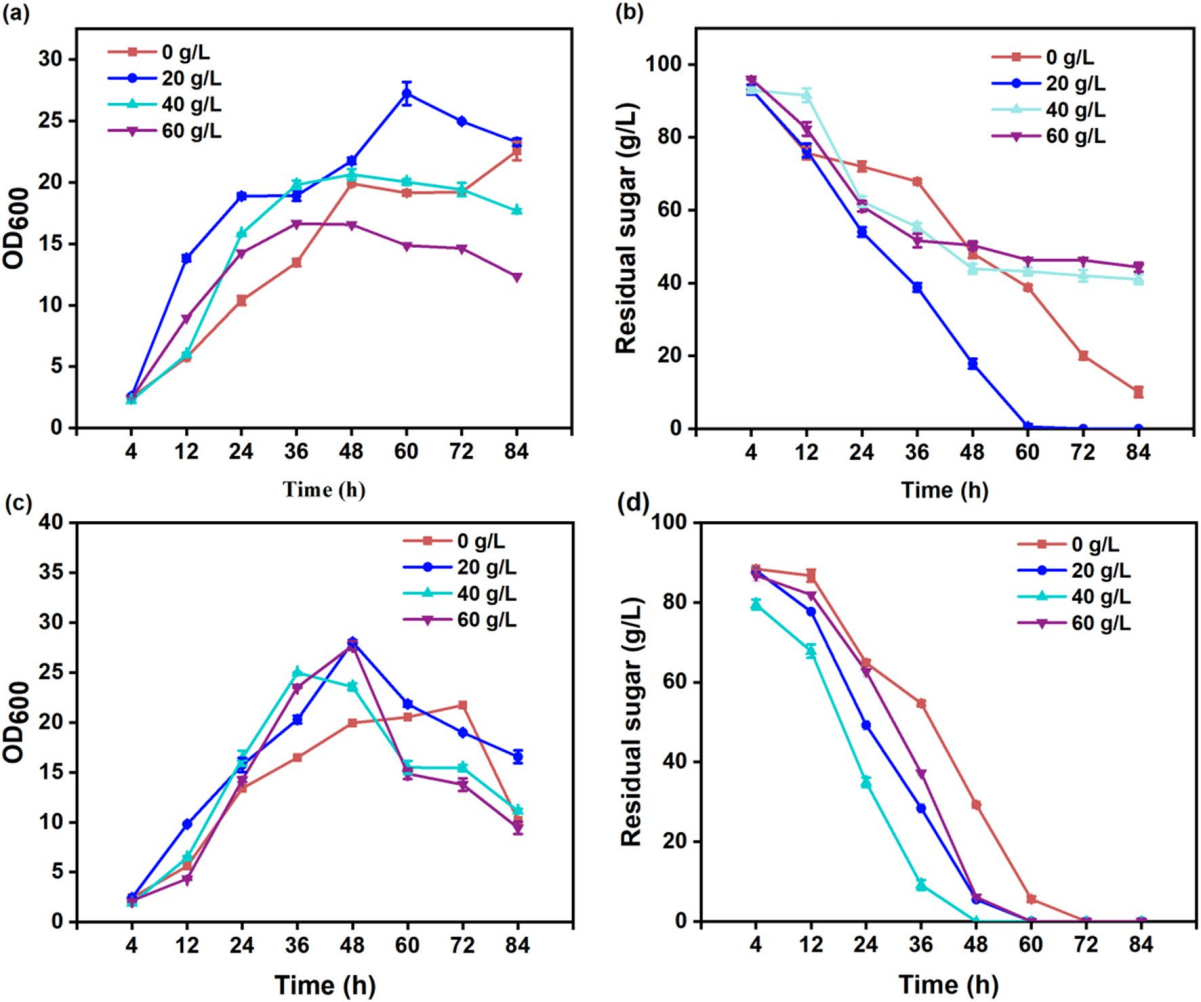
Effect of different concentrations of acetoin stress on cell growth and glucose consumption of *B. subtilis* 168-pMA5 (**a**, **b**) and *B. subtilis* 168-*phaCBA* (**c**, **d**)

A comprehensive assessment was conducted to elucidate the influence of varying acetoin concentrations on the growth of *B. subtilis* 168-pMA5 and *B. subtilis* 168-*phaCBA*. Figure 1 illustrates that *B. subtilis* 168-*phaCBA* exhibited significantly elevated growth rates across all tested acetoin concentrations (0, 40, 60, and 80 g/L) in comparison to *B. subtilis* 168-pMA5. Notably, *B. subtilis* 168*-phaCBA* demonstrated a remarkable capacity to tolerate 80 g/L of acetoin, sustaining growth, while *B. subtilis* 168-pMA5 exhibited an inability to thrive under these conditions. To comprehensively investigate the metabolic and gene transcript responses of *B. subtilis* 168-*phaCBA* to acetoin stress, we conducted additional experiments assessing the impact of different acetoin concentrations (0, 20, 40, and 60 g/L) on the growth and sugar consumption during acetoin fermentation. Our findings revealed that acetoin supplementation impeded the growth of *B. subtilis* during acetoin fermentation. Notably, the utilization of glucose by the recombinant strain was significantly hindered when the acetoin concentration exceeded 40 g/L in the medium (as depicted in Fig. 2a, b). In pursuit of a comprehensive understanding of the transcriptional and metabolomic responses to acetoin stress, we focused on the severe acetoin stress group with a concentration of 60 g/L acetoin for subsequent analyses. This deliberate selection enabled a detailed exploration of the intricate regulatory mechanisms and metabolic shifts within *B. subtilis* 168-pMA5 and *B. subtilis* 168-*phaCBA* under high acetoin stress conditions.

### Effects of acetoin stress on gene expression in *B. subtilis* 168-pMA5 and *B. subtilis* 168-*phaCBA*

**Fig. 3:**
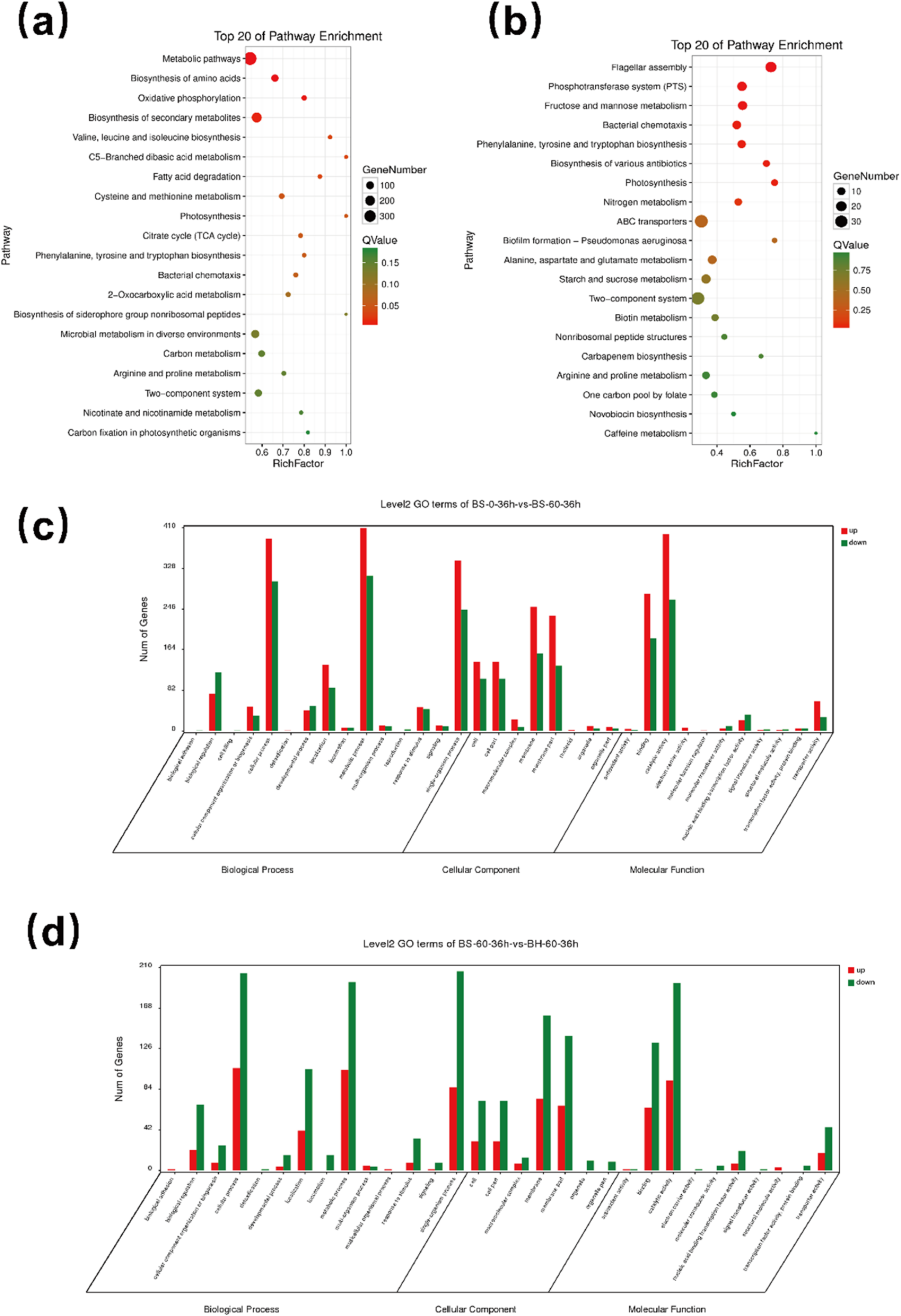
Differentially expressed genes (DEGs) GO classification analysis and KEGG classification analysis of *B. subtilis* 168-pMA5 and *B. subtilis* 168-*phaCBA* under acetoin stress. **a** Identification of enriched DEGs by KEGG database in the 60 g/L acetoin treatment group (*B. subtilis* 168-pMA5) and 0 g/L acetoin control group (*B. subtilis* 168-pMA5). **b** KEGG enriched pathways of DEGs after acetoin stress between *B. subtilis* 168-pMA5 and *B. subtilis* 168-*phaCBA*. **c** GO analysis of DEGs in the 60 g/L acetoin treatment group (*B. subtilis* 168-pMA5) and 0 g/L acetoin control group (*B. subtilis* 168-pMA5). **d** GO analysis of DEGs after acetoin stress between *B. subtilis* 168-pMA5 and *B. subtilis* 168-*phaCBA*

To elucidate the tolerance mechanism of *B. subtilis* 168-*phaCBA* under acetoin stress, we examined the differences in gene expression between *B. subtilis* 168-pMA5 and *B. subtilis* 168-*phaCBA* under acetoin stress. We also compared the gene expression variations between *B. subtilis* 168-pMA5 and *B. subtilis* 168-*phaCBA* under acetoin stress. Differential gene expression was analyzed using edgeR software, focusing on genes with a False Discovery Rate (FDR) of less than 0.05 and an absolute log2 fold change (|log2FC|) greater than 1. The results indicated that under acetoin stress, *B. subtilis* 168-pMA5 showed 1774 significantly differentially expressed genes, with 1003 genes up-regulated and 771 genes down-regulated. Gene Ontology (GO) analysis, categorized these genes into three main groups: biological processes, cellular components, and molecular functions. In the biological process category, up-regulated and down-regulated genes were primarily associated with cellular processes, metabolic processes, and single-organism processes. In the cellular component category, the genes were concentrated in cells, cell parts, membranes, and membrane parts. In the molecular function category, the up-regulated and down-regulated genes were primarily involved in binding and catalytic activity (as illustrated in Fig. 3c).

Kyoto Encyclopedia of Genes and Genomes (KEGG) analysis unveiled significant enrichment in metabolic pathways (Fig. 3a), These pathways encompassed biosynthesis of amino acids, oxidative phosphorylation, biosynthesis of secondary metabolites, amino acid metabolism (valine, leucine, isoleucine biosynthesis, cysteine, and methionine), C5-branched dibasic acid metabolism, fatty acid degradation, photosynthesis, TCA cycle, phenylalanine, tyrosine, and tryptophan biosynthesis, 2-oxo carboxylic acid metabolism, biosynthesis of siderophore group nonribosomal peptides, microbial metabolism in diverse environments, carbon metabolism, arginine and proline metabolism, two-component system, nicotinate and nicotinamide metabolism, carbon fixation in photosynthetic organisms, β-alanine metabolism, tryptophan metabolism, glycolysis/gluconeogenesis, biosynthesis of various antibiotics, among others (Additional file 1: Table S1).

Comparatively, *B. subtilis* 168-*phaCBA* exhibited 863 significantly different genes compared to the control strain *B. subtilis* 168-pMA5. Among these, 294 genes were significantly up-regulated, while 569 genes were significantly down-regulated (Fig. 3d). In the Biological Process category, both up-regulated and down-regulated genes in *B. subtilis* 168-*phaCBA* were mainly associated with cellular processes, localization, metabolic processes, and signal-organism processes. In the Cellular Component category, these genes were predominantly related to cells, cell parts, membranes, and membrane parts. In the Molecular Function category, both up-regulated and down-regulated genes were centered on binding and catalytic activity. KEGG enrichment analysis identified the 20 pathways with the highest enrichment degree (Fig. 3b). The significantly altered DEGs in *B. subtilis* 168-*phaCBA* were enriched in pathways such as flagellar assembly, phosphotransferase system (PTS), fructose and mannose metabolism, bacterial chemotaxis, phenylalanine, tyrosine, and tryptophan biosynthesis, biosynthesis of various antibiotics, photosynthesis, nitrogen metabolism, ABC transporters, biofilm formation, and alanine, aspartate, and glutamate metabolism (Additional file 1: Table S2).

### Metabolomics analysis of intracellular metabolite changes in *B. subtilis* 168-pMA5 and *B. subtilis *168-*phaCBA* under acetoin stress

**Fig. 4:**
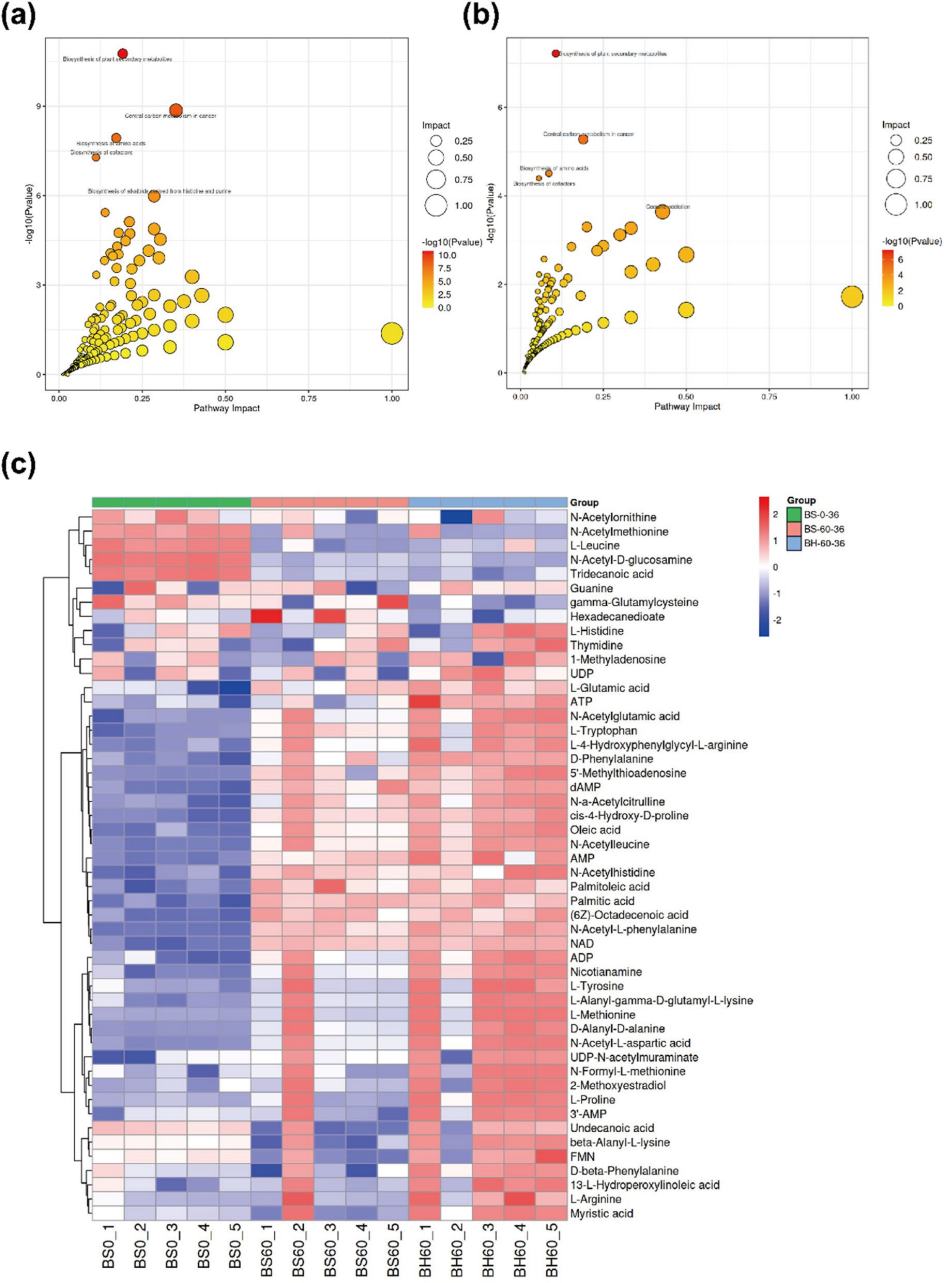
Metabolic pathway analysis of significantly differential metabolites and cluster analysis of significantly differential metabolite contents after acetoin stress treatment. **a** Metabolic pathway analysis of significantly differential metabolites in the 60 g/L acetoin treatment group (*B. subtilis* 168-pMA5) and 0 g/L acetoin control group (*B. subtilis* 168-pMA5). **b** Metabolic pathway analysis of significantly differential metabolites in acetoin stress between *B. subtilis* 168-pMA5 and *B. subtilis* 168-*phaCBA*. **c** Heatmap obtained by hierarchical clustering analysis of differential metabolites between *B. subtilis* 168-pMA5 (0 g/L, 60 g/L acetoin stress) and *B. subtilis* 168-*phaCBA* (60 g/L acetoin stress)

To unravel the physiological changes associated with the heterologous expression of *phaCBA* in *B. subtilis* 168 under acetoin stress, we employed MetaboAnalyst to scrutinize the KEGG pathway enrichment of differential metabolites in two conditions: *B. subtilis* 168-pMA5 under 0 g/L and 60 g/L acetoin, and the comparison between *B. subtilis* 168-pMA5 and *B. subtilis* 168-*phaCBA* under 60 g/L acetoin stress (Fig. 4a, b). The scatter plot in Fig. 4a illustrates the impact factor, reflecting significant metabolic pathway changes in *B. subtilis* 168-pMA5 under severe acetoin stress. These changes encompassed pathways such as the biosynthesis of plant secondary metabolites, central carbon metabolism in cancer, biosynthesis of amino acids, biosynthesis of cofactors, and biosynthesis of alkaloids derived from histidine and purine, among others. Similarly, the metabolic pathway changes between *B. subtilis* 168-pMA5 and *B. subtilis* 168-*phaCBA* under severe acetoin stress were comparable, as shown in Fig. 4b. Clustered heat maps in Fig. 4c depict alterations in intracellular metabolites in *B. subtilis* 168-pMA5 (0 g/L and 60 g/L acetoin) and *B. subtilis* 168-*phaCBA* (60 g/L acetoin). These heat maps highlight significant changes in intracellular amino acid fractions, membrane fatty acid groups, and energy metabolism in both *B. subtilis* 168-pMA5 and *B. subtilis* 168-*phaCBA* under severe acetoin stress. Notably, when compared with *B. subtilis* 168-pMA5, *B. subtilis* 168-*phaCBA* exhibited a significant increase in the content of l-tryptophan, l-tyrosine, l-leucine, l-threonine, l-methionine, l-glutamic acid, l-proline, d-phenylalanine, and l-arginine in the intracellular amino acid components. The content of saturated fatty acids, such as myristic acid, hexadecanedioate, and tridecanoic acid, decreased significantly, while the content of unsaturated fatty acids, including oleic acid, palmitoleic acid, and undecanoic acid, increased. Additionally, the cofactors UDP, NDP, FMN, ADP, and AMP also significantly increased. These findings suggest that *B. subtilis* 168-*phaCBA* exhibited enhanced viability under severe acetoin stress.

### Effect of acetoin on fatty acid composition of cell membranes in *B. subtilis *168-pMA5 and *B. subtilis* 168-*phaCBA*

According to previous reports in the literature, microorganisms facing various unfavorable environmental stresses alter the fatty acid composition of cell membranes, thereby enhancing coping capacity. To further elucidate the tolerance mechanism of *B. subtilis* 168-*phaCBA* to high acetoin concentrations, we investigated the effects of acetoin (0 and 60 g/L) on the fatty acid composition of the cell membranes in both *B. subtilis* 168-pMA5 and *B. subtilis* 168-*phaCBA*. In the absence of acetoin stress, the contents of membrane fatty acid fractions in *B. subtilis* 168-pMA5 and *B. subtilis* 168-*phaCBA* were significantly different. Under acetoin stress, the proportions of saturated fatty acids C16:0 in the cell membrane fatty acids of *B. subtilis* 168-pMA5 underwent substantial changes, reducing from 80.06 to 11.62%. Meanwhile, the proportions of saturated fatty acids i-C15:0, C15:0, and C17:0 increased from 0.8%, 3.88%, and 1.04% to 10.67%, 35.57%, and 11.1%, respectively. The unsaturated fatty acid C22:1, did not change significantly. Simultaneously, the cell membrane fatty acid fractions in *B. subtilis* 168-*phaCBA* also exhibited alterations, with the most significant change observed in the unsaturated fatty acid C22:1, increasing from 12.72 to 26.06%.

The results, as presented in Table 1, revealed that in *B. subtilis* 168-*phaCBA*, the total unsaturated fatty acids (TUFA) and the ratio of unsaturated fatty acids to saturated fatty acids (U/S) gradually increased, while the total saturated fatty acids (TSFA) decreased with increasing acetoin concentration. In contrast, *B. subtilis* 168-pMA5 did not exhibit an increase in the content of unsaturated fatty acids or the U/S ratio with acetoin stress. Notably, the TUFA content in *B. subtilis* 168-*phaCBA* was 29.6% and 137.6% higher than that in *B. subtilis* 168-pMA5 in the presence of 0 and 60 g/L acetoin, respectively. Moreover, the U/S ratios in *B. subtilis* 168-*phaCBA* were 33.3% and 191.6% higher than those in *B. subtilis* 168-pMA5 at 0 and 60 g/L acetoin concentration, respectively.

**Table 1:** Effect of acetoin concentration on fatty acid composition of *Bacillus subtilis* membrane

Fatty acid composition^c^ (%)	*B. subtilis*168*-*pMA5 (0 g/L)	*B. subtilis*168*-phaCBA* (0 g/L)	*B. subtilis*168*-*pMA5 (60 g/L)	*B. subtilis*168*-phaCBA* (60 g/L)
C14: 0	0.13 ± 0.006	1.43 ± 0.045^a^	0.69 ± 0.096^a^	2.32 ± 0.101^a,b^
i-C15:0	0.80 ± 0.600	8.58 ± 1.055^a^	10.67 ± 0.858^a^	11.49 ± 0.240^a^
C15:0	3.88 ± 0.501	32.22 ± 0.737^a^	35.57 ± 0.467^a^	26.03 ± 0.570^a,b^
iC16:0	0.31 ± 0.005	5.82 ± 0.157^a^	2.35 ± 0.068^a^	8.28 ± 0.200^a,b^
C16:0	80.06 ± 2.08	12.51 ± 0.179^a^	11.62 ± 0.118^a^	5.56 ± 0.219^a,b^
C17:0	1.04 ± 0.011	8.83 ± 1.210^a^	11.10 ± 1.175^a^	11.38 ± 0.920^a,b^
C18:2	1.14 ± 0.015	NA	NA	NA
C18:1	0.63 ± 0.010	NA	NA	NA
C18: 0	0.86 ± 0.050	1.71 ± 0.072^a^	1.77 ± 0.115^a^	0.55 ± 0.021^a,b^
C22:1	11.12 ± 1.015	16.72 ± 1.47^a^	10.97 ± 0.297^a^	26.06 ± 0.165^a,b^
14-C17:0	NA	11.04 ± 0.302^a^	12.68 ± 0.206^a^	8.11 ± 0.856^a,b^
16-C17:0	NA	1.14 ± 0.025^a^	2.54 ± 0.200^a^	0.22 ± 0.021^a,b^
TSFA	87.48 ± 0.591	83.28 ± 1.467^a^	88.98 ± 0.296^a^	73.94 ± 0.169^a,b^
TUFA	12.90 ± 1.040	16.72 ± 1.469^a^	10.97 ± 0.297^a^	26.06 ± 0.165^a,b^
TPUFA	1.15 ± 0.015	0^a^	0^a^	0^a^
U/S	0.15	0.2	0.12	0.35

*TSFA* total saturated fatty acids, *TUFA* total unsaturated fatty acids, *TPUFA* total polyunsaturated fatty acid, *U/S* total unsaturated fatty acids/total saturated fatty acids

^a^Indicates a statistically significant difference in the same fatty acid groups *B. subtilis*-*phaCBA* (0 g/L), *B. subtilis*-pMA5 (60 g/L) and *B. subtilis*-*phaCBA* (60 g/L) compared with *B. subtilis*-pMA5 (0 g/L) (*p* < 0.05)

^b^Indicates a statistically significant difference in the same fatty acid in *B. subtilis*-*phaCBA* (60 g/L) compared with *B. subtilis*-pMA5 (60 g/L) (*p* < 0.05)

^c^Data are the means ± standard deviations (SDs) from three parallel experiments

### Metabolic and transcriptional differences analysis

**Fig. 5:**
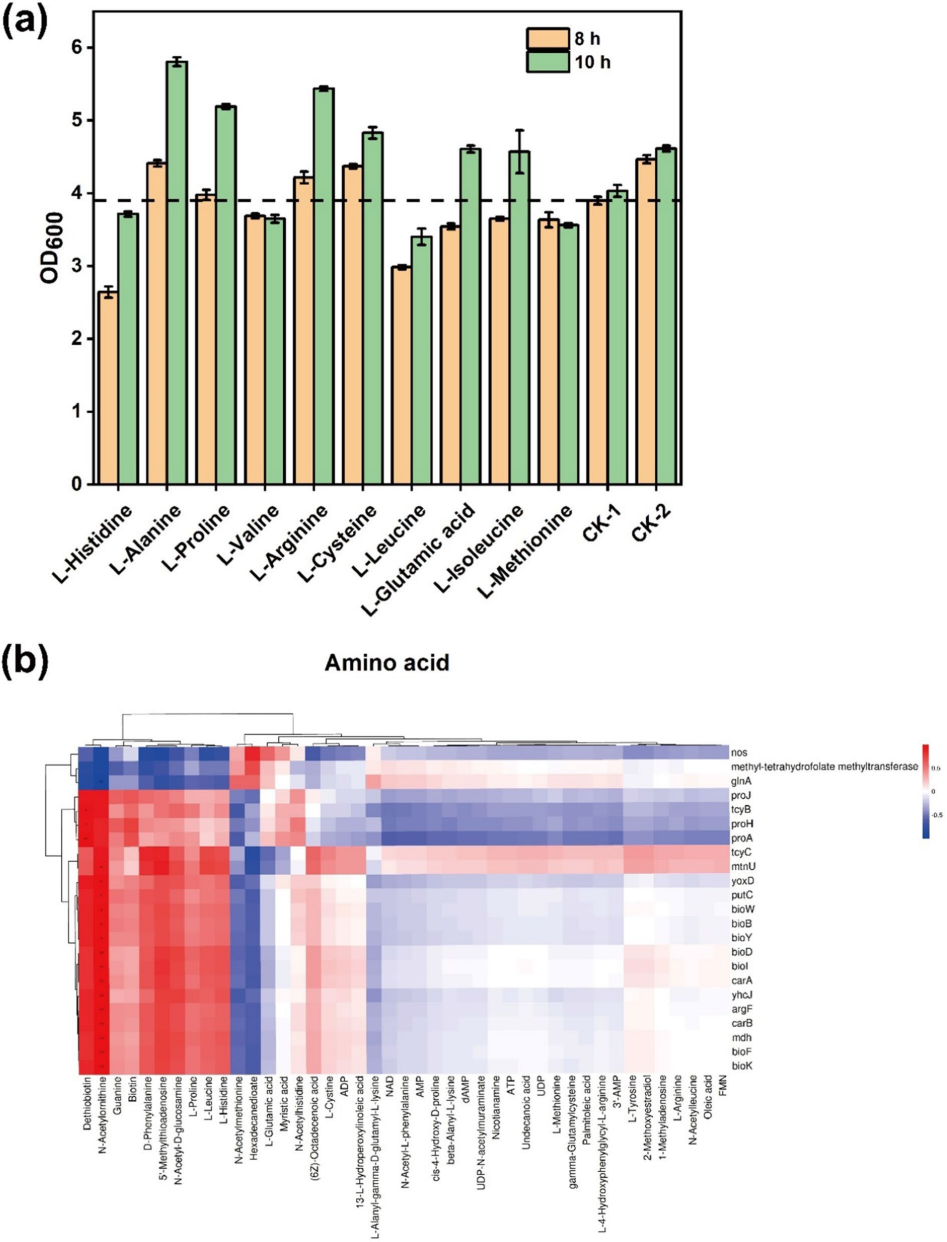
**a** Effect of exogenous addition of different amino acids (l-histidine, l-alanine, l-proline, l-valine, l-arginine, l-cysteine, l-leucine, l-glutamic acid, l-isoleucine, l-methionine) on the growth of *B. subtilis* 168 under acetoin stress. **b** Differential expression results for metabolites and related transcripts. Horizontal coordinates are associated metabolites, vertical coordinates are transcript names, red indicates positive correlation, and blue indicates negative correlation

To delve into the distinctions between *B. subtilis* 168-pMA5 and *B. subtilis* 168-*phaCBA*, we pinpointed 23 differentially expressed genes (DEGs) concurrently regulated in both comparison groups out of the 43 differentially expressed metabolites (DEMs). The regulated levels of these DEGs are depicted in Fig. 5b. Notably, genes involved in dethiobiotin and biotin synthesis, such as *bioY*, *bioB*, *bioD*, *bioW*, *bioI*, *bioF*, and *bioK*, exhibited up-regulation in the *B. subtilis* 168-*phaCBA* group (Additional file 3: Table S4). Metabolites, including *N*-acetylornithine, guanine, d-phenylalanine, 5′-methythioadenosine, *N*-acetyl-d-glucosamine, l-proline, l-leucine, and l-histidine, demonstrated significant correlations with these biosynthetic genes. Conversely, a marked negative correlation was observed with metabolites like *N*-acetylmethionine and hexadecanedioate. Among the DEGs, the expression of *tcyC* (cystine ABC transporter) and *mtnU* (ketoglutaramate omega-amidase) increased by 1.3-fold and 1.75-fold, respectively. These genes are associated with the synthesis of l-cysteine and (6z)-Octadecenoic acid, contributing to *Bacillus* resistance. In contrast, there was a significant decrease in the expression of genes like nitric-oxide synthase (*nos*), methyl-tetrahydrofolate methyltransferase (*yxjH*), and glutamine synthetase (*glnA*). These genes were positively correlated with the synthesis of the saturated fatty acid hexadecanedioate and negatively correlated with the synthesis of l-proline, unsaturated fatty acid (6z)-Octadecenoic acid, and l-cysteine.

To comprehend the impact of intracellular amino acids on *B. subtilis* under acetoin stress, we supplemented the acetoin stress medium with various amino acids. After 8 h of fermentation incubation, it was found that exogenous supplementation with l-alanine (OD_600_ = 4.41), l-arginine (OD_600_ = 4.21) and l-cysteine (OD_600_ = 4.37), significantly increased the biomass of *B. subtilis* 168 compared to other amino acids and the blank control CK-1 (OD_600_ = 3.90). Continued fermentation up to 10 h revealed that the biomass of *B. subtilis* was higher than the blank control CK-1 with exogenous supplementation of l-alanine, l-proline, l-arginine, l-cysteine, l-glutamic acid, and l-isoleucine. The results demonstrated that exogenous supplementation of l-alanine, l-proline, l-cysteine, l-arginine, l-glutamic acid, and l-isoleucine partially alleviated acetoin stress (Fig. 5a). Considering that the earlier release of acetoin stress inhibition during the growth of *B. subtilis* is more favorable for product synthesis, supplementation with l-alanine, l-arginine, and l-cysteine was the most effective.

### Shake flask batch fermentation culture of recombinant strains

**Fig. 6:**
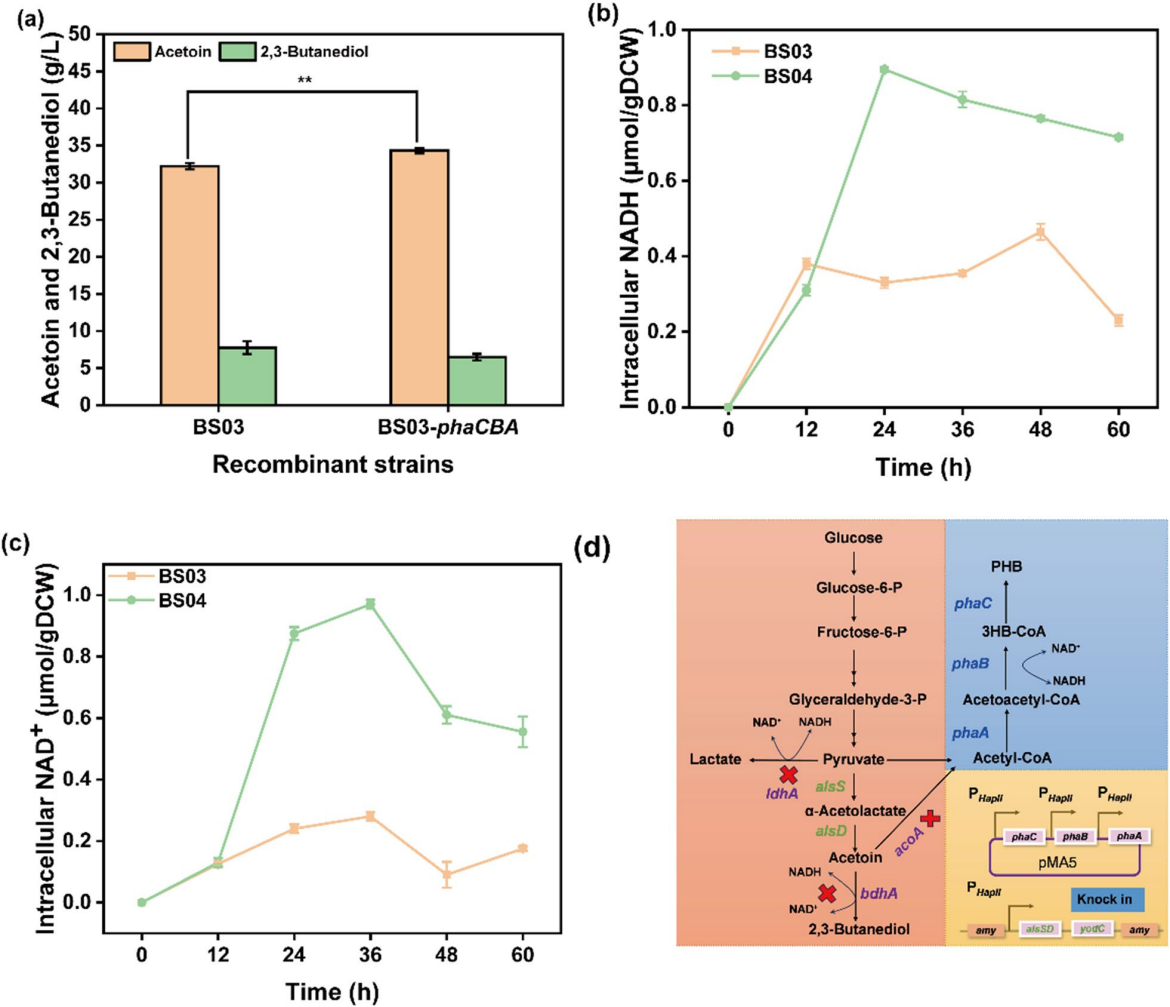
**a** Fermentation experiments with recombinant strains BS03 and BS03-*phaCBA* of acetoin **significance code: *P* < 0.01. **b**, **c** Effects of heterologous expression *phaCBA* on intracellular NADH and NAD^+^ concentrations. **d** Overview of the production of acetoin from glucose by *B. subtilis* engineered bacteria. Blue words represent genes expressed on plasmid pMA5. Green words represent genes integratively expressed. Purple words represent knockout genes

The aerobic fermentation results for the recombinant *B. subtilis* strains indicated that strain BS03 (*B. subtilis* 168Δ*bdhA*Δ*acoA*Δ*LdhA*, integrated expression of *alsSD*-*yodC* under P_*HapII*_ at the amy locus) increased the acetoin concentration by 6.48% to approximately 34.3 g/L compared to BS03-*phaCBA*. Additionally, the concentration of the by-product 2,3-Butanediol decreased by 16.52% (Fig. 6a). To understand these differences, changes in intracellular cofactors (NAD^+^/NADH) in the recombinant strains were analyzed. The results showed that the concentrations of intracellular cofactors NAD^+^ and NADH were higher in BS03-*phaCBA* compared to BS03 throughout the entire fermentation cycle (Fig. 6b, c). This suggests that the expression of genes related to the metabolic pathway of acetoin was stronger in BS03-*phaCBA*. One possible reason for this observation is that the knockdown of lactate dehydrogenase (*ldhA*) and acetoin reductase (*bdhA*) led to NADH accumulation. Both of these enzymes are NADH-dependent, and acetoacetyl-CoA derived from *Halomonas bluephagenesis* TD01 has a significant preference for NADH, reducing the toxic effects of NADH accumulation on the cells [21, 22].

### Fed-batch fermentation

**Fig. 7:**
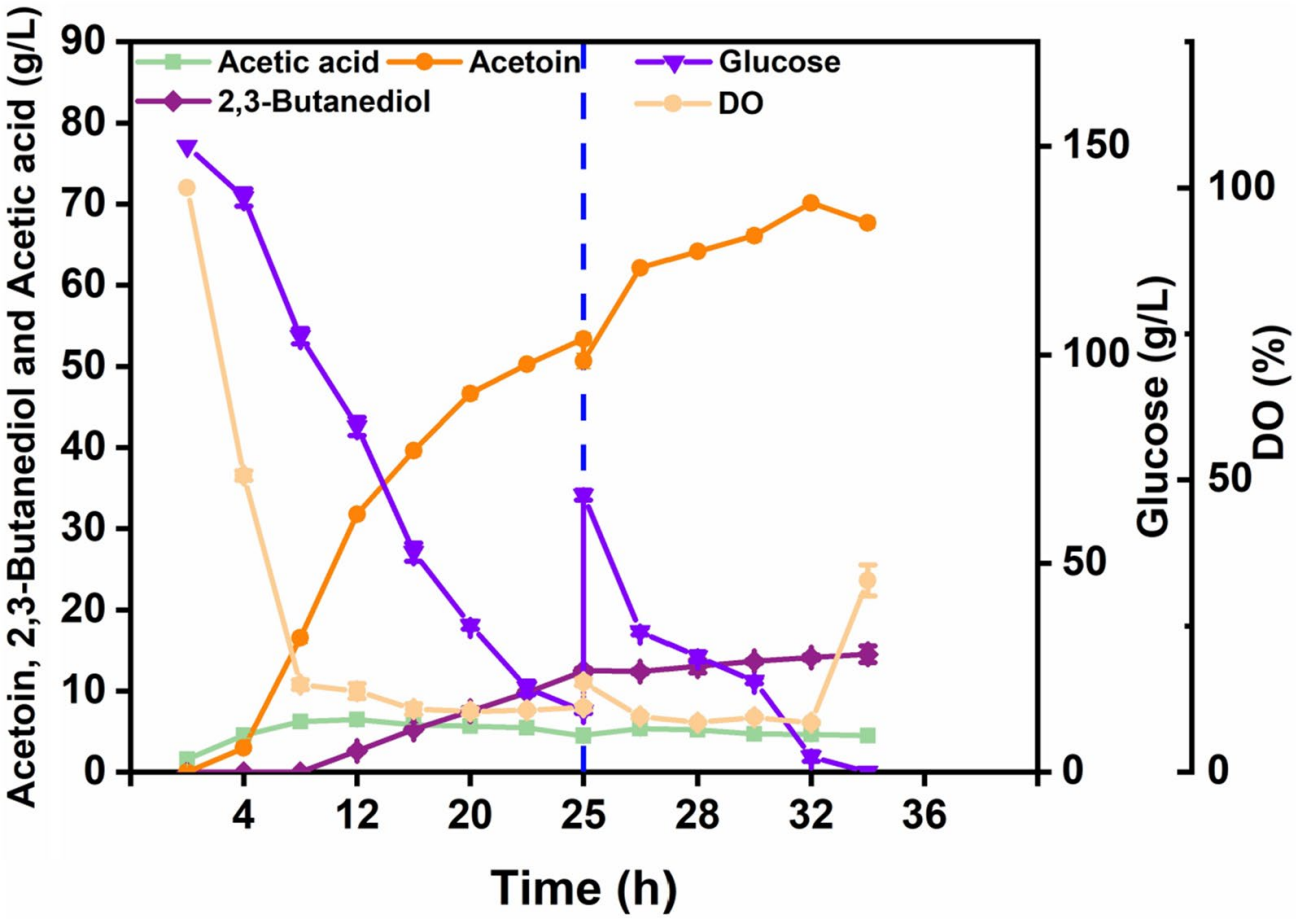
Batch fermentation of recombinant strain BS03-*phaCBA* in a 7.5 L bioreactor for the production of acetoin

To assess the acetoin production capacity of recombinant strain BS03-*phaCBA*, the fed-batch fermentation was conducted in a 7.5 L fermenter. As shown in Fig. 7, at 25 h of fermentation, the residual sugar content and acetoin concentration were 15.4 g/L and 50.66 g/L, respectively. During this period, the dissolved oxygen increased from 11.4 to 15.6% compared to 24 h. Generally, a high oxygen supply favors acetoin formation and decreases 2,3-butanediol final titer [23]. Therefore, after disposable batch feeding, the speed is increased to 800 rpm. The highest yield of 70.14 g/L was achieved at 34 h of fermentation.

## Discussion

In our tolerance experiments, we observed significant growth inhibition of *B. subtilis* 168 when the acetoin concentration reached 40 g/L or higher. To gain insights into the genetic and metabolic changes occurring under acetoin stress (60 g/L), transcriptomics and metabolomics analyses were conducted, revealing alterations in 24 metabolic pathways. Some of these pathways play crucial roles in resisting acetoin stress and maintaining normal cellular functions.

Oxidative phosphorylation: Genes related to oxidative phosphorylation showed up-regulation, suggesting that *B. subtilis* requires enhanced oxidative phosphorylation and increased respiration to maintain intra- and extracellular homeostasis under acetoin stress (Additional file 2: Table S3) [24]. Photosynthesis and fatty acid degradation: Genes involved in photosynthesis and fatty acid degradation were significantly up-regulated. The induction of these operons may be related to glucose starvation, affecting glucose transport and utilization, leading to sugar starvation, spore formation, and increased expression of genes in the fatty acid degradation pathway [25]. Two-component system: Genes such as *dnaA*, *kinC*, and *kapB*, associated with the two-component system, showed increased expression. These genes are related to DNA replication and spore formation. The up-regulation of these genes reflects the strain’s stress response to the virulence of *B. subtilis* 168 under acetoin stress. Efflux Pumps and CAMP Repulsion: Genes associated with bacitracin efflux (*bceA*, *vraD*) and CAMP repulsion (e.g., *dltA*, *dltB*, *dltC*, *dltD*, *dltE*) exhibited a significant reduction in expression (Additional file 2: Table S3). Efflux pumps play a crucial role in transporting toxic substances out of the cell [26]. A high concentration of acetoin influx, coupled with reduced efflux pump activity, can lead to damage to macromolecules like proteins and DNA. This can inhibit cell growth or even cause cell lysis and death [27]. C5-Branched-dibasic acid metabolism: Genes associated with C5-branched dibasic acid metabolism, linked to acetoin synthesis, exhibited significant reduction, explaining the inability to sustain acetoin production at later fermentation stages. Valine, leucine, and isoleucine biosynthesis: Overall down-regulation of genes associated with valine, leucine, and isoleucine biosynthesis was observed. Branched-chain amino acids have been shown to play a role in maintaining cell membrane fluidity and integrity, possibly contributing to *B. subtilis* tolerance [28]. Overall, these findings provide insights into various molecular mechanisms and pathways involved *B. subtilis* 168’s response to acetoin stress. Understanding these mechanisms is crucial for developing strategies to improve acetoin tolerance and production in this strain.

In this study, the heterologous expression of *phaCBA* in *B. subtilis* 168 was found to significantly improve the tolerance to acetoin. Transcriptomic analyses identified key changes in gene expression contributing to this enhanced tolerance. Down-regulation of flagellar assembly and bacterial chemotaxis: A notable finding was the significant decrease in the expression of genes related to flagellar assembly and bacterial chemotaxis. Under stressful conditions, inhibiting flagellar assembly might be an energy-saving strategy, allowing the bacterium to allocate resources more efficiently to other survival mechanisms [29, 30]. Similarly, reduced chemotaxis activity helps the cell adapt to its environment and allocate resources more wisely under stressful conditions. This aligns with previous studies that have shown down-regulation of flagellar biosynthesis in response to stress, suggesting a conserved strategy among bacteria [31, 32]. Up-regulation of transporter proteins: Several transporter protein genes, such as molybdate (*modA*, *modB*), osmoprotectant (*opuBC*, *opuBB*, *opuBA*), biotin (*bioY*), and zinc/manganese/iron (II), were significantly up-regulated in the ABC transporters system (Additional file 3: Table S4). Biotin, in particular, is essential for some enzymes involved in fatty acid synthesis [33]. Its deficiency can lead to a reduced proportion of unsaturated fatty acids in cell membranes, which could impact the cell’s defenses against stressors [34, 35]. Studies have shown that the addition of biotin uptake genes can improve the growth rate and solvent yield in other bacterial strains, emphasizing the beneficial role of up-regulating biotin transporter protein genes for enhancing tolerance to acetoin in *B. subtilis* [36]. Understanding these changes in gene expression provides valuable insights into the mechanisms underlying acetoin tolerance improvement in *B. subtilis* 168 with heterologous expression of *phaCBA*. This knowledge is essential for the development of strategies aimed at further optimizing acetoin production and tolerance in this strain. Up-regulating biotin transporter protein genes is beneficial for enhancing *B. subtilis*’ tolerance to acetoin. Up-regulation of cystine and d-methionine transporter proteins: The observed up-regulation of transporter protein genes for cystine (*tcyB*, *tcyC*) and d-methionine (*metQ*) in the ABC transporters system suggests an important role in acetoin tolerance. Cysteine is known to play a role in essential cellular functions such as the assembly of iron-sulfur clusters, which can mitigate oxidative damage [37]. The positive effects of l-cysteine on reductases and peroxidases involved in scavenging reactive oxygen species (ROS) align with the notion that acetoin stress can increase intracellular ROS production, inducing damage and apoptosis [38, 39]. By enhancing the transport of cystine and d-methionine, the strain’s tolerance to acetoin was increased, potentially due to improved antioxidant defenses. Up-regulation of energy metabolism-related metabolites: Intracellular metabolomic analysis showed that the contents of ADP, ATP, FMN, and 3′-AMP in *B. subtilis* 168-*phaCBA* were significantly up-regulated compared to *B. subtilis* 168-pMA5 under acetoin stress (Fig. 4c). This suggests an enhanced energy metabolism in response to acetoin stress. Increased energy production is necessary to support the enhanced tolerance capacity of the strain. Fatty acid composition alterations and improved acetoin tolerance: The influence of acetoin stress on the fatty acid composition of microbial membranes is highlighted in the study. Under high concentrations of acetoin stress, the proportion of unsaturated fatty acids in *B. subtilis* increased, and the ratio of unsaturated to saturated fatty acids (U/S) was significantly higher in *B. subtilis* 168-*phaCBA* compared to *B. subtilis* 168-pMA5. This alteration, attributed to the expression of *phaCBA*, enhances the strain’s tolerance to acetoin by maintaining the fluidity and integrity of the cell membrane [40–42]. The increased content of unsaturated fatty acids aligns with findings in other studies where modification of membrane fatty acid fractions contributed to improved tolerance to various stresses [43]. These study findings indicate a connection between altering membrane fatty acid composition and structure and the enhancement of organic solvent tolerance. Enhanced tolerance through altered amino acids: The significant changes in intracellular amino acids, such as l-proline, l-glutamic acid, l-histidine, l-methionine, l-arginine, l-tyrosine, and l-leucine further contribute to the enhanced tolerance to acetoin in *B. subtilis* 168-*phaCBA*. The addition of exogenous amino acids, including l-alanine, l-proline, l-glutamic acid, l-arginine, and l-isoleucine, alleviated the stress imposed by acetoin. These amino acids have been shown to enhance stress tolerance in various organisms. For instance, arginine addition can protect yeast from ethanol damage by maintaining the integrity of the cell wall and cytoplasmic membranes [44]. Proline and glutamic acid accumulation can increase the tolerance of *B. subtilis* to butanol and maintain normal biological function under stress conditions [45]. Methionine can enhance the antioxidant stress capacity of the strain by converting it to cysteine [46, 47]. The review article by Saini et al. extensively discussed the amino acids linked to ethanol stress tolerance in yeast cells [48]. Among these, isoleucine, methionine, phenylalanine, proline, and tryptophan were found effective in mitigating the stress induced by ethanol. In our study, both isoleucine and proline demonstrated equal efficacy in alleviating the stress caused by acetoin. Combinatorial analysis of metabolites and gene expressions: The combinatorial analysis of significantly changed metabolites confirmed the relevance of certain metabolites and related gene expressions in enhancing *B. subtilis*’ ability to overcome acetoin stress. These include metabolites such as l-proline, l-cysteine, (6z)-Octadecenoic acid, and biotin, which were up-regulated. Conversely, the content of the saturated fatty acid hexadecanedioate and its expression were significantly down-regulated. The changes in metabolites and gene expressions collectively contribute to the enhanced tolerance of *B. subtilis* to acetoin stress. In conclusion, the combination of increased unsaturated fatty acids, altered amino acid profiles, and metabolite changes, in response to the heterologous expression of *phaCBA*, enhances the tolerance of *B. subtilis* 168 to acetoin stress. This study provides valuable insights into the mechanisms underlying stress tolerance in microbial strains and may have practical implications for industrial applications. Understanding these mechanisms could contribute to the development of more resilient microbial strains for various biotechnological processes.

Practical implications for industrial applications: The findings of this study hold practical implications for industrial applications, where enhancing strain tolerance to toxic products can significantly increase the fermentation yields of target products. Previous studies, such as those on *E. coli* overexpressing specific genes to overcome butanol tolerance, have demonstrated the effectiveness of enhancing strain tolerance for improved fermentation yields. The introduction of chaperones and chaperone-like proteins has also shown promise in enhancing tolerance to solvents and increasing the synthesis of specific products [49, 50]. In the case of *B. subtilis* 168-*phaCBA*, the study suggests that the heterologous expression of *phaCBA* can be a valuable strategy for improving acetoin tolerance and production. The construction of the engineered acetoin-producing bacterial strain BS03-*phaCBA* achieved an impressive acetoin yield of 70.14 g/L in fed-batch fermentation, highlighting its potential for biotechnological and industrial applications. Nevertheless, as evident from recent studies, there are critical avenues for refinement to enhance acetoin production in this strain. As identified in the literature, the synthesis of intracellular polyhydroxybutyrate (PHB) enhances acetoin tolerance but concurrently imposes a metabolic burden on the acetoin synthesis pathway. Consequently, a precise metabolic balance is imperative to maximize acetoin production while mitigating the burdens associated with PHB synthesis. Achieving this balance involves sophisticated genetic engineering to fine-tune metabolic pathways and enzyme activities. In conclusion, while our study has made significant strides in acetoin production, it also underscores the untapped potential for optimization. Further research and engineering efforts are warranted to unravel the full potential of acetoin production in *B. subtilis* 168-*phaCBA*, offering opportunities for advancements in biotechnological applications.

## Conclusion

In summary, the introduction of *phaCBA* expression into *B. subtilis* 168 led to significant alterations in the cell membrane’s fatty acid composition and influenced the intracellular uptake and synthesis of amino acids and biotin. Consequently, this modification enhanced the strain’s tolerance to acetoin. Our findings underscore the importance of synthesizing long-chain unsaturated fatty acids, along with supplementing specific amino acids such as l-alanine, l-proline, l-glutamic acid, l-arginine, and l-isoleucine, in alleviating acetoin-induced stress. The metabolomic and transcriptomic analyses conducted in this study have revealed a diverse array of metabolites that contribute to acetoin tolerance. These insights pave the way for future investigations and the rational design of more resilient hosts for acetoin production. Overall, this study provides valuable contributions to the understanding of microbial responses to acetoin stress and sets the stage for further advancements in strain engineering for enhanced acetoin production. The identified strategies and mechanisms may have broader implications for improving stress tolerance in microbial hosts for various industrial applications.

## Material and methods

### Strains and plasmids

*Escherichia coli* DH5α was employed for plasmid amplification. *B. subtilis* 168 and BS03 (*B. subtilis* 168Δ*bdhA*Δ*acoA*Δ*LdhA*, integrated expression of *alsSD*-*yodC* under P_*HapII*_ at the amy locus) were maintained in our laboratory. BS03-*phaCBA* was constructed in this study. The gene sequences of PHB synthesis pathway-related genes *phaC* (derived from *Cupriavidus necator*, GeneID: 57643537), *phaB* (derived from *Halomonas bluephagenesis* TD01, LOCUS: WP_009724067) and *phaA* (derived from *Cupriavidus necator*, GeneID: 57643536) was obtained from the National Center of Biotechnology Information (NCBI) database, and codon-optimized and synthesized de novo by GenScript Biotech Corporation. These genes were expressed with the plasmid pMA5 in both *B. subtilis* 168 and BS03-*phaCBA*. The recombinant plasmid was introduced into *B. subtilis* using the Spizizen method [51].

### Medium and culture conditions

The recombinant strain was streaked on LB solid medium (10 g/L tryptone, 5 g/L yeast powder, 10 g/L NaCl, 20 g/L agar powder) and then incubated overnight in a 37 °C incubator. A loop of colonies from the LB solid medium was collected and inoculated into 50 mL/250 mL LB liquid medium, followed by incubation at 37 °C, with shaking at 160 rpm for 12 h to generate seed cultures. The seeds culture was then transferred at 5% (v/v) into 250 mL shake flasks containing 50 mL of fermentation medium (100 g/L glucose, 5 g/L yeast powder, 15 g/L corn steep powder, 2 g/L Urea, pH6.8). Microaerobic and aerobic fermentation were performed at 37 ℃ with shaking at 160 rpm and 220 rpm, respectively. The acetoin tolerance test involved the following steps. Recombinant strains, cultured under microaerobic conditions for 36 h, were centrifuged (6000 rpm, 10 min), washed three times with PBS (pH7.4), and inoculated into LB liquid medium containing various acetoin concentrations 0, 40, 60, and 80 g/L). Initial OD_600_ was maintained between 1.8 and 2.1, and the cultures were then incubated at 37 ℃ and 220 rpm for 10 h. To assess the impact of acetoin addition on bacterial growth during fermentation, recombinant strains were cultured in acetoin fermentation medium for 4 h at 37 ℃ and 160 rpm. Subsequently, acetoin was added to achieve final concentrations of 0 g/L, 20 g/L, 40 g/L, 60 g/L, and 80 g/L, respectively. Biomass measurements were taken at various time points up to the conclusion of the 84-h fermentation. All analytical reagents were purchased from Nanjing Wanqing Chemical Glass Wear & Instrument Co. Ltd.

### Transcriptional analysis

Cells of both *B. subtilis* 168-pMA5 and *B. subtilis* 168-*phaCBA* were collected after 36 h of cultivation under conditions with and without 60 g/L acetoin. Collected cells were washed three times with sterile water, frozen in liquid nitrogen, and stored at − 80 ℃ for subsequent RNA-seq analyses. Total RNA was extracted using the TRIzol-based method (Life Technologies, CA, USA). RNA degradation and potential contamination were assessed by 1% agarose gels. RNA purity was determined by OD260/OD280, and OD260/OD230 ratios using the NanoPhotometer® spectrophotometer (IMPLEN, CA, USA). To enrich for mRNA and deplete rRNA, 1 µg of total RNA was processed using the Illumina MRZB12424 Ribo-Zero rRNA Removal Kit (Bacteria) (Illumina, San Diego, CA, USA). The cDNA, DNA, and small RNA libraries were subsequently subjected to sequencing on the Illumina platform by Genedenovo Biotechnology Co., Ltd (Guangzhou, China). To analyze gene expression levels, the data were normalized using the fragments Per Kilobase of Transcript per Million (FPKM) mapped reads method. Normalization accounts for differences in gene lengths and sequencing data quantity. The identification of differentially expressed genes (DEGs) was carried out using the edgeR package (http://www.r-project.org/), with fold changes ≥ 2 and a false discovery rate-adjusted *P* value (q value) < 0.05 as the criteria for significance DEGs were subsequently subjected to an enrichment analysis of Gene Ontology (GO) functions and Kyoto Encyclopedia of Genes and Genomes (KEGG) pathways. A threshold of q value < 0.05 was applied to identify significantly enriched functional categories and pathways.

### LC–MS-based metabolomics analysis

Cells were collected after cultivating 36 h of cultivation under different acetoin concentrations (0 g/L, 60 g/L). For each sample, broth equivalent to 200 mg of cells was collected by centrifugation at 6000 rpm for 10 min at 4 °C. Collected pellets were immediately frozen using liquid nitrogen and stored at − 80 °C until further analysis. Metabolite extraction, liquid chromatography-mass spectrometry (LC–MS) analysis, annotation and data preprocessing were carried out by Shanghai Personal Biotechnology Co., Ltd (Shanghai, China). The LC analysis was performed on a Vanquish UHPLC System (Thermo Fisher Scientific, USA). Chromatography was conducted using an ACQUITY UPLC HSS T3 column (2.1 × 100 mm, 1.8 µm) (Waters, Milford, MA, USA) maintained at 40 ℃. The flow rate and injection volume were set at 0.3 mL/min and 2 μL, respectively. For LC-ESI (+)-MS analysis, the mobile phases consisted of (B2) 0.1% formic acid in acetonitrile (v/v) and (A2) 0.1% formic acid in water (v/v) [52]. Mass spectrometric detection was performed using an Orbitrap Exploris 120 mass spectrometer (Thermo Fisher Scientific, USA) with an ESI ion source. Simultaneous MS1 and MS/MS (Full MS-ddMS2 mode, data-dependent MS/MS) acquisition was employed [53]. Key parameters included sheath gas pressure (40 arb), aux gas flow (10 arb), spray voltage (3.50 kV and − 2.50 kV for ESI (+) and ESI (−), respectively), capillary temperature (325 °C), MS1 range (m/z 100–1000), MS1 resolving power (60,000 FWHM), number of data-dependent scans per cycle, MS/MS resolving power (15,000 FWHM), normalized collision energy (30%), and dynamic exclusion time (automatic). Differential metabolites were subjected to pathway analysis using MetaboAnalyst, combining results from powerful pathway enrichment analysis with pathway topology analysis [54]. The identified metabolites in metabolomics were then mapped to KEGG pathways for biological interpretation of higher-level systemic functions. Metabolites and corresponding pathways were visualized using the KEGG Mapper tool.

### Membrane fatty acid analysis

Lipids from *B. subtilis* 168-pMA5 and *B. subtilis* 168-*phaCBA* cells were extracted and methylated following the MIDI method [55]. After freeze–drying, the tested bacterial strain was added to a screw-capped test tube. Then, 5 mL of 0.4 M KOH-CHO_3_H was added and saponified at 60 °C for 1 h. After cooling, 4 mL of HCl-CH_3_OH (V/V = 1:9) was added and the mixture was bathed at 60 °C for 20 min. Upon cooling, 3% NaCl (w/v) was added, followed by fatty acid extraction using n-hexane. Gas Chromatography–Mass Spectrometry (GC–MS) analyses were conducted using a 7890A GC and 5975C MSD (Agilent Technologies, Waldbronn, Germany) and samples were analyzed using a DB-5MS capillary column. Helium was used as the carrier gas at a flow rate of 1 mL/min, the injection volume was set to 1 μL, and the detector temperature was set to 260 °C. The injector temperature and column oven temperature were both set to 280 °C. Mass spectrometry conditions for GC–MS analysis included ionization in EI mode with an electron energy of 70 eV, ion source temperature of 230 °C, and quadrupole temperature of 150 °C [56].

### Growth analysis of *B. subtilis* 168 with exogenous amino acid addition

*Bacillus subtilis* 168 cells were initially cultured at 37 °C for 12 h. Subsequently, they were inoculated into the LB liquid medium containing 40 g/L acetoin. The effects of various amino acids on cell growth were studied by adding these 0.5% (w/v) amino acids (l-histidine, l-alanine, l-proline, l-valine, l-arginine, l-cysteine, l-leucine, l-glutamic acid, l-isoleucine, l-methionine) to LB liquid medium containing 40 g/L acetoin. The control groups consisted of LB liquid medium with 40 g/L acetoin without amino acid supplementation (control group 1, CK-1) and LB liquid medium without the addition of acetoin and amino acids (control group 2, CK-2).

### Fed-batch fermentation in 7.5-L fermenter

Fed-batch fermentations were conducted in a 7.5-L fermenter with a working volume of 3 L. The operational parameters included a pH of 6.5, a temperature of 37 °C, and a rotation speed of 600 rpm. The inoculum volume was set at 5% (v/v), and the aeration ratio was 2 vvm (air volume per culture volume per minute). The initial glucose concentration in the medium was 150 g/L. When the glucose level dropped below 20 g/L, a 200 mL feed medium, containing 500 g/L glucose and 60 g of yeast powder dissolved in 100 mL sterile water, was introduced into the fermenter. The rotation speed was increased from 600 to 800 rpm.

### Analysis methods

Acetoin, acetic acid and 2,3-butanediol were quantified using liquid chromatography with an Agilent 1290 Infinity instrument (Agilent Technologies, Waldbronn, Germany). An Aminex HPX-87H column was used, with an injection volume of 20 μL. The mobile phase consisted of 5 mM H_2_SO_4_, and the flow rate was set at 0.6 mL/min. The column temperature was maintained at 65 °C, and a refractive index detector was employed [57]. Biomass was determined using a spectrophotometer (756S, China) at 600 nm. The cell dry weight was calculated using the empirical formula 1 OD_600_ = 0.352 DCW (g/L) [58]. Intracellular concentrations of NADH and NAD^+^ were determined using the Beyotime Biotechnology (Shanghai, China) NAD^+^/NADH Assay Kit (WST-8 method) following the manufacturer’s instructions [59].

### Supplementary Information

**Additional file1: Table S1.** Pathway enrichment of *B. subtilis* 168-pMA5 under acetoin stress. **Table S2.** Pathway enrichment of *B. subtilis* 168-pMA5 and *B. subtilis* 168-*phaCBA* under acetoin stress.

**Additional file 2: Table S3.** All the gene difference analysis results of *B. subtilis* 168-pMA5 under acetoin stress.

**Additional file 3: Table S4.** All the gene difference analysis results of *B. subtilis* 168-pMA5 and *B. subtilis* 168-*phaCBA* under acetoin stress.

## Data Availability

The obtained data will be available from the corresponding author upon reasonable request.

## Abbreviations

PHB: Polyhydroxyalkanoates
LC–MS: Liquid chromatography mass spectrometry
TUFA: Total unsaturated fatty acids
TSFA: Total saturated fatty acids
TPUFA: Total polyunsaturated fatty acid
KEGG: Kyoto Encyclopedia of Genes and Genomes
DEGs: Differentially expressed genes
DEMs: Differentially expressed metabolites
*B. subtilis* 168: *Bacillus subtilis* 168
PBS: Phosphatic buffer solution
OD: Optical density
