# Supplementary material for: Analysis of heterologous expression of phaCBA promotes the acetoin stress response mechanism in Bacillus subtilis using transcriptomics and metabolomics approaches
Source: Microb Cell Fact. 2024 Feb 21;23:58. doi: 10.1186/s12934-024-02334-z (PMC10880289; doi:10.1186/s12934-024-02334-z)
Supplement: Supplementary file 1 — Additional file1: Table S1. Pathway enrichment of B. subtilis 168-pMA5 under acetoin stress. Table S2. Pathway enrichment of B. subtilis 168-pMA5 and B. subtilis 168-phaCBA under acetoin stress. [file 12934_2024_2334_MOESM1_ESM.docx]

***Supplementary materials***

**Table S1 Pathway enrichment of *B. subtilis* 168-pMA5 under acetoin stress**

|  | **Pathway** | **Candidate genes with pathway annotation (633)** | **All genes with pathway annotation (1282)** | **Pvalue** | **Qvalue** | **Pathway ID** |
| --- | --- | --- | --- | --- | --- | --- |
| 1 | Metabolic pathways | 388 (61.3%) | 714 (55.69%) | 0.000042 | 0.003559 | ko01100 |
| 2 | Biosynthesis of amino acids | 82 (12.95%) | 124 (9.67%) | 0.000057 | 0.003559 | ko01230 |
| 3 | Oxidative phosphorylation | 28 (4.42%) | 35 (2.73%) | 0.000162 | 0.006686 | ko00190 |
| 4 | Biosynthesis of secondary metabolites | 192 (30.33%) | 334 (26.05%) | 0.000353 | 0.010947 | ko01110 |
| 5 | Valine, leucine and isoleucine biosynthesis | 12 (1.9%) | 13 (1.01%) | 0.00142 | 0.030038 | ko00290 |
| 6 | C5-Branched dibasic acid metabolism | 9 (1.42%) | 9 (0.7%) | 0.001695 | 0.030038 | ko00660 |
| 7 | Fatty acid degradation | 14 (2.21%) | 16 (1.25%) | 0.001696 | 0.030038 | ko00071 |
| 8 | Cysteine and methionine metabolism | 34 (5.37%) | 49 (3.82%) | 0.003109 | 0.047591 | ko00270 |
| 9 | Photosynthesis | 8 (1.26%) | 8 (0.62%) | 0.003454 | 0.047591 | ko00195 |
| 10 | Citrate cycle (TCA cycle) | 18 (2.84%) | 23 (1.79%) | 0.004182 | 0.051861 | ko00020 |
| 11 | Phenylalanine, tyrosine and tryptophan biosynthesis | 16 (2.53%) | 20 (1.56%) | 0.004769 | 0.053759 | ko00400 |
| 12 | Bacterial chemotaxis | 19 (3%) | 25 (1.95%) | 0.005748 | 0.059395 | ko02030 |
| 13 | 2-Oxocarboxylic acid metabolism | 21 (3.32%) | 29 (2.26%) | 0.00943 | 0.089944 | ko01210 |
| 14 | Biosynthesis of siderophore group nonribosomal peptides | 6 (0.95%) | 6 (0.47%) | 0.014317 | 0.12681 | ko01053 |
| 15 | Microbial metabolism in diverse environments | 109 (17.22%) | 192 (14.98%) | 0.015936 | 0.131737 | ko01120 |
| 16 | Carbon metabolism | 58 (9.16%) | 97 (7.57%) | 0.021053 | 0.149276 | ko01200 |
| 17 | Arginine and proline metabolism | 19 (3%) | 27 (2.11%) | 0.021326 | 0.149276 | ko00330 |
| 18 | Two-component system | 74 (11.69%) | 127 (9.91%) | 0.021669 | 0.149276 | ko02020 |
| 19 | Nicotinate and nicotinamide metabolism | 11 (1.74%) | 14 (1.09%) | 0.025143 | 0.164093 | ko00760 |
| 20 | Carbon fixation in photosynthetic organisms | 9 (1.42%) | 11 (0.86%) | 0.029275 | 0.181502 | ko00710 |
| 21 | beta-Alanine metabolism | 7 (1.11%) | 8 (0.62%) | 0.032103 | 0.189219 | ko00410 |
| 22 | Tryptophan metabolism | 12 (1.9%) | 16 (1.25%) | 0.033571 | 0.189219 | ko00380 |
| 23 | Glycolysis / Gluconeogenesis | 25 (3.95%) | 39 (3.04%) | 0.043555 | 0.234818 | ko00010 |
| 24 | Biosynthesis of various antibiotics | 8 (1.26%) | 10 (0.78%) | 0.049776 | 0.257178 | ko00998 |

|  | Pathway | Candidate genes with pathway annotation (296) | All genes with pathway annotation (1282) | Pvalue | Qvalue | Pathway ID |
| --- | --- | --- | --- | --- | --- | --- |
| 1 | Flagellar assembly | 24 (8.11%) | 33 (2.57%) | 0 | 0 | ko02040 |
| 2 | Phosphotransferase system (PTS) | 16 (5.41%) | 29 (2.26%) | 0.000156 | 0.008144 | ko02060 |
| 3 | Fructose and mannose metabolism | 15 (5.07%) | 27 (2.11%) | 0.000228 | 0.008144 | ko00051 |
| 4 | Bacterial chemotaxis | 13 (4.39%) | 25 (1.95%) | 0.001404 | 0.036591 | ko02030 |
| 5 | Phenylalanine, tyrosine and tryptophan biosynthesis | 11 (3.72%) | 20 (1.56%) | 0.001842 | 0.036591 | ko00400 |
| 6 | Biosynthesis of various antibiotics | 7 (2.36%) | 10 (0.78%) | 0.002052 | 0.036591 | ko00998 |
| 7 | Photosynthesis | 6 (2.03%) | 8 (0.62%) | 0.002647 | 0.040464 | ko00195 |
| 8 | Nitrogen metabolism | 9 (3.04%) | 17 (1.33%) | 0.006821 | 0.091234 | ko00910 |
| 9 | ABC transporters | 36 (12.16%) | 118 (9.2%) | 0.031741 | 0.37736 | ko02010 |
| 10 | Biofilm formation - Pseudomonas aeruginosa | 3 (1.01%) | 4 (0.31%) | 0.040458 | 0.398641 | ko02025 |
| 11 | Alanine, aspartate and glutamate metabolism | 13 (4.39%) | 35 (2.73%) | 0.040982 | 0.398641 | ko00250 |

**Table S2 Pathway enrichment of *B. subtilis 168-pMA5 and B. subtilis 168-phaCBA* under acetoin stress**
